# Supplementary material for: Association between Wait Time for Transthoracic Echocardiography and 28-Day Mortality in Patients with Septic Shock: A Cohort Study
Source: J Clin Med. 2022 Jul 16;11(14):4131. doi: 10.3390/jcm11144131 (PMC9321017; doi:10.3390/jcm11144131)
Supplement: Supplementary file 1 [file jcm-11-04131-s001.zip › Supplementary Table S1.pdf]

Supplementary Table S1. Relationship between TTE wait time as a continuous variable and 28-day mortality in two-piece-wise Cox regression model.

**Wait time（as a continuous variable）**	**HR (95% CI)**	**P value**
**⩽10 hours**	1.05 (1.01, 1.10)	0.013
**＞10 hours**	0.99 (0.98, 1.01)	0.349
**Log likelihood ratio test**		0.015
