# Supplementary material for: Association between Wait Time for Transthoracic Echocardiography and 28-Day Mortality in Patients with Septic Shock: A Cohort Study
Source: J Clin Med. 2022 Jul 16;11(14):4131. doi: 10.3390/jcm11144131 (PMC9321017; doi:10.3390/jcm11144131)
Supplement: Supplementary file 1 [file jcm-11-04131-s001.zip › Supplementary Table S2.pdf]

Supplementary Table S2. Baseline characteristics of participants

**Covariate**	**All patients**	**Unmatched Patients**			**Propensity-Score–Matched Patients**		
		**Time of TTE**			**Time of TTE**		
		**Early (≤10h)**	**Delayed (＞10h)**	**P value**	**Early (≤10h)**	**Delayed (＞10h)**	**P value**
N	3264	544	2720		532	532	
Age(years) ， mean (SD)	67.8 ± 16.0	68.9 ± 15.0	67.6 ± 16.1	0.078	68.7 ± 15.1	68.8 ± 15.0	0.890
Male, sex, no.(%)	1758 (53.9)	310 (57.0)	1448 (53.2)	0.109	301 (56.6)	296 (55.6)	0.757
Weight（kg）， mean (SD)	82.2 ± 25.2	83.1 ± 24.6	82.0 ± 25.3	0.353	83.3 ± 24.6	83.9 ± 28.1	0.724
**Vital Signs，mean (SD)**							
Heart rate (bpm)	94.3 ± 21.8	92.2 ± 21.9	94.7 ± 21.8	**0.012**	92.4 ± 22.1	92.0 ± 21.0	0.798
MAP (mmHg)	75.5 ± 18.6	74.2 ± 18.1	75.8 ± 18.7	0.069	74.3 ± 18.2	74.7 ± 17.0	0.730
Respiratory rate (bpm)	20.1 ± 6.7	18.7 ± 6.8	20.4 ± 6.6	**＜0.001**	18.7 ± 6.8	18.9 ± 6.7	0.592
**Laboratory tests，mean (SD)**							
WBC (×109)	14.9 ± 11.0	15.0 ± 8.7	14.9 ± 11.4	0.753	15.0 ± 8.8	14.8 ± 8.9	0.606
Hemoglobin (g/L)	10.6 ± 2.7	10.5 ± 2.3	10.6 ± 2.7	0.143	10.5 ± 2.3	10.6 ± 2.1	0.577
Platelet (×1012)	216.0 ± 127.2	206.3 ± 112.6	217.9 ± 129.9	**0.034**	207.4 ± 112.6	209.5 ± 119.9	0.774
Sodium (mmol/L)	138.2 ± 5.5	137.4 ± 4.5	138.3 ± 5.7	**＜0.001**	137.5 ± 4.5	137.6 ± 5.3	0.858
Potassium (mmol/L)	4.2 ± 0.9	4.4 ± 0.9	4.2 ± 0.9	**＜0.001**	4.3 ± 0.9	4.3 ± 1.1	0.354
Chloride (mmol/L)	106.4 ± 6.9	106.3 ± 5.8	106.5 ± 7.1	0.550	106.3 ± 5.8	106.4 ± 6.6	0.856
Bicarbonate (mmol/L)	21.2 ± 5.1	21.3 ± 4.6	21.2 ± 5.2	0.537	21.3 ± 4.7	21.2 ± 4.7	0.922
Creatinine (mg/dL)	1.7 ± 1.5	1.6 ± 1.2	1.7 ± 1.6	0.052	1.6 ± 1.2	1.6 ± 1.2	0.860
BUN (mg/dL)	33.6 ± 24.7	30.8 ± 20.6	34.2 ± 25.4	**0.003**	31.0 ± 20.7	30.4 ± 19.7	0.655
BNP (tested) , n (%)	73 (2.2)	8 (1.5)	65 (2.4)	0.184	8 (1.5)	7 (1.3)	0.795
Troponin (tested) , n (%)	1307 (40.0)	243 (44.7)	1064 (39.2)	**0.018**	238 (44.7)	225 (42.3)	0.421
Creatinine kinase (tested) , n (%)	1847 (56.6)	310 (57.0)	1537 (56.6)	0.879	306 (57.5)	305 (57.3)	0.951
Lactate (tested) , n (%)	2670 (81.8)	479 (88.1)	2191 (80.7)	**＜0.001**	467 (87.8)	466 (87.6)	0.926
**Severity of illness，mean (SD)**							
SAPS II	48.4 ± 15.2	47.7 ± 14.3	48.5 ± 15.4	0.205	47.7 ± 14.3	47.9 ± 14.4	0.783
APS III	60.4 ± 23.9	57.1 ± 23.4	61.0 ± 24.0	**＜0.001**	57.2 ± 23.4	57.8 ± 22.5	0.708
SOFA	7.9 ± 3.7	7.8 ± 3.4	7.9 ± 3.7	0.712	7.8 ± 3.4	7.7 ± 3.5	0.701
OASIS	38.9 ± 8.7	38.1 ± 8.2	39.1 ± 8.8	**0.010**	38.2 ± 8.2	38.3 ± 7.8	0.757
**Interventions , n (%)**							
Ventilator use	2450 (75.1)	466 (85.7)	1984 (72.9)	**＜0.001**	454 (85.3)	457 (85.9)	0.793
Sedative Use(1st 24h)	2236 (68.5)	439 (80.7)	1797 (66.1)	**＜0.001**	427 (80.3)	436 (82.0)	0.481
RRT(1st 24 h)	208 (6.4)	29 (5.3)	179 (6.6)	0.276	29 (5.5)	28 (5.3)	0.892
**Comorbidity disease (%)**							
CHF	1209 (37.0)	277 (50.9)	932 (34.3)	**＜0.001**	266 (50.0)	263 (49.4)	0.854
AFIB	1147 (35.1)	262 (48.2)	885 (32.5)	**＜0.001**	252 (47.4)	257 (48.3)	0.759
CAD	803 (24.6)	243 (44.7)	560 (20.6)	**＜0.001**	231 (43.4)	238 (44.7)	0.666
Renal	485 (14.9)	95 (17.5)	390 (14.3)	0.061	91 (17.1)	94 (17.7)	0.808
COPD	510 (15.6)	74 (13.6)	436 (16.0)	0.155	74 (13.9)	77 (14.5)	0.792
Stroke	238 (7.3)	54 (9.9)	184 (6.8)	**0.010**	51 (9.6)	62 (11.7)	0.274

Covariate entries in bold have p values < 0.05.

Bpm: beat per minute, CAD: coronary heart disease, CHF: congestive heart failure, AFIB: atrial fibrillation, COPD: chronic

obstructive pulmonary disease, MAP: mean arterial pressure, RRT: renal replace treatment, SAPS: simplified acute physiology score, APS: acute physiology score, SOFA: sequential organ failure assessment, OASIS: oxford acute severity of illness score, RRT: renal replace treatment, WBC: white blood count, BUN: blood urea nitrogen, BNP: brain natriuretic peptide.
