# Supplementary material for: Association between Wait Time for Transthoracic Echocardiography and 28-Day Mortality in Patients with Septic Shock: A Cohort Study
Source: J Clin Med. 2022 Jul 16;11(14):4131. doi: 10.3390/jcm11144131 (PMC9321017; doi:10.3390/jcm11144131)
Supplement: Supplementary file 1 [file jcm-11-04131-s001.zip › Supplementary Table S3.pdf]

Supplementary Table S3. The results of sensitivity analysis.

**TTE time groups**	**HR (95% CI)**	**P value**
**⩽10h**	Ref.	
**＞10, ⩽40h**	1.26 (1.01, 1.58)	0.043
**>40h**	0.99 (0.77, 1.26)	0.915
**No TTE**	1.89 (1.51, 2.38)	<0.0001
