# Supplementary figures and images for: Association between Wait Time for Transthoracic Echocardiography and 28-Day Mortality in Patients with Septic Shock: A Cohort Study
Source: J Clin Med. 2022 Jul 16;11(14):4131. doi: 10.3390/jcm11144131 (PMC9321017; doi:10.3390/jcm11144131)

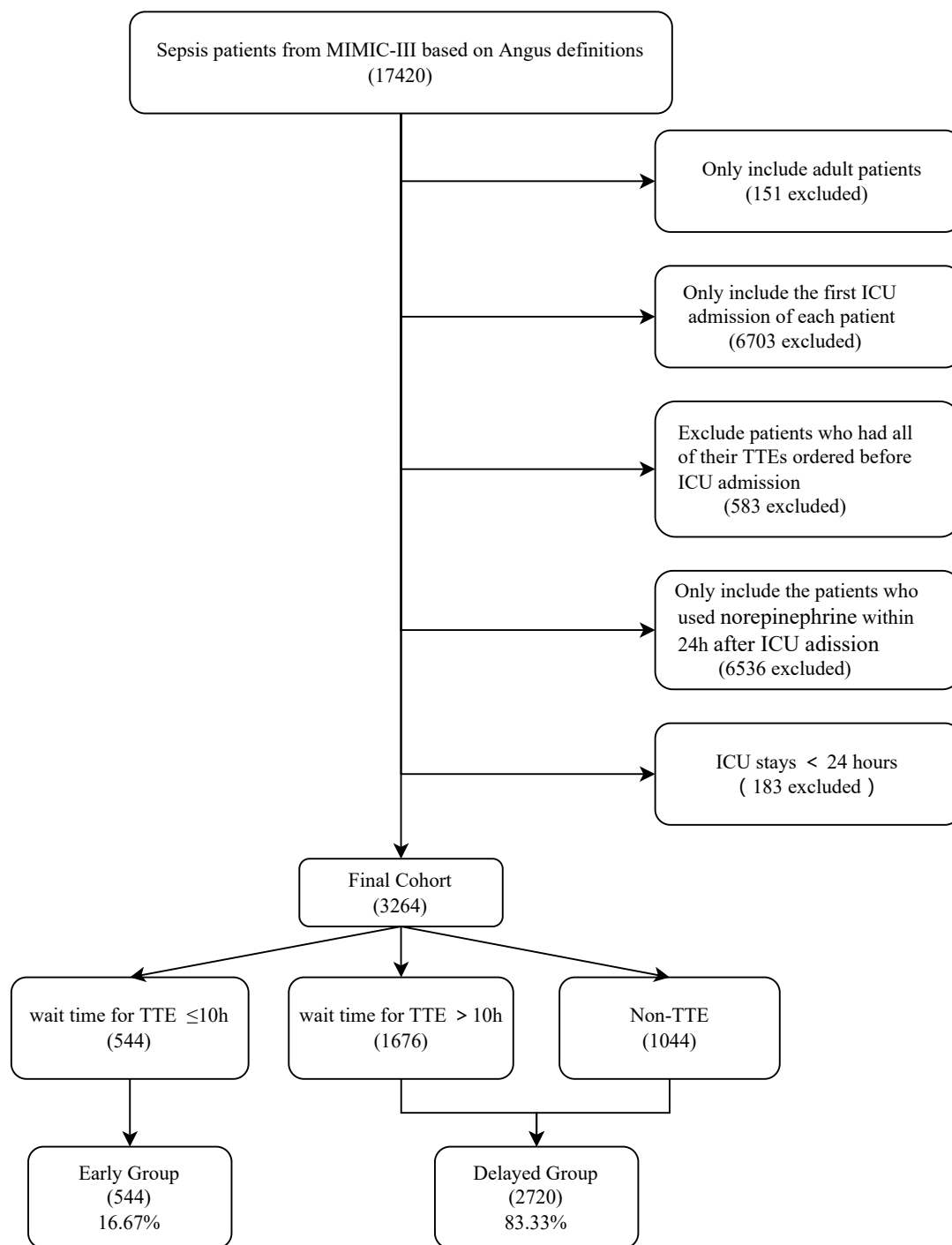

Supplementary Figure S1. Flowchart of the study cohort.

Supplement: Supplementary file 1 [file jcm-11-04131-s001.zip › Supplementary Figure S1. flowchart.pdf]
